# A new species of *Arostrilepis* from *Ellobius tancrei* (Rodentia: Cricetidae) in Mongolia

**DOI:** 10.1017/S0031182022000294

**Published:** 2022-05

**Authors:** Altangerel T. Dursahinhan, Daniel R. Brooks, Sebastian Botero-Cañola, Scott L. Gardner

**Affiliations:** 1 Harold W. Manter Laboratory of Parasitology, University of Nebraska State Museum, University of Nebraska-Lincoln, W-529 Nebraska Hall, Lincoln, NE 68588-0514, USA; 2 Department of Ecology and Evolutionary Biology, University of Toronto (Emeritus), Toronto, Ontario, Canada; 3 Institute for Evolution, Centre for Ecological Research, Karolina ut 29, Budapest H-1113, Hungary

**Keywords:** *Arostrilepis*, Baitag Bogd, *Ellobius tancrei*, helminth parasites, Mongolia, subterranean mammal

## Abstract

Cestodes of the genus *Arostrilepis* Mas-Coma and Tenora 1997 have a Holarctic distribution with 16 species occurring among 28 species of mostly arvicoline hosts. The type species of the genus is *Arostrilepis horrida* (von Linstow, 1901), described initially as *Taenia horrida* von Linstow, 1901, from murine rodents in Lower Saxony (Niedersachsen), Germany. Here we report the first helminth parasite from the mole-vole, *Ellobius tancrei*, in Mongolia which is the first subterranean rodent known to be infected with *Arostrilepis* in the Palearctic. In addition, we describe a new species: *Arostrilepis batsaikhani* n. sp. which most closely resembles *A. microtis* Gulyaev and Chechulin 1997, differing from this species with a genetic distance of about 4% (using cytochrome-b) and by having distinctly large cirrus spines, testes that are larger and fill the whole segment measured anterior–posterior and larger eggs.

## Graphical abstract

**Figure:**
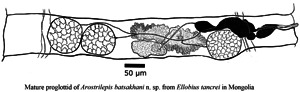

## Introduction

This paper reports on the continuing work by our team of mammalogists and parasitologists who participated in the Mongolian Vertebrate Parasite Project (MVPP). The field work of the MVPP first started in 1999 and funding from the United States National Science Foundation was secured to start the main expeditionary field research in the summer of 2009 with fieldwork continuing through 2012. The overall goal of the MVPP was to discover, describe and document the distribution of vertebrates and their parasites in the south-central and south-western areas of the Gobi Desert/steppe-grasslands and eastern Altai Mountains in Mongolia (see summary in Tinnin *et al.*, 2008).

**Fig. 1.**
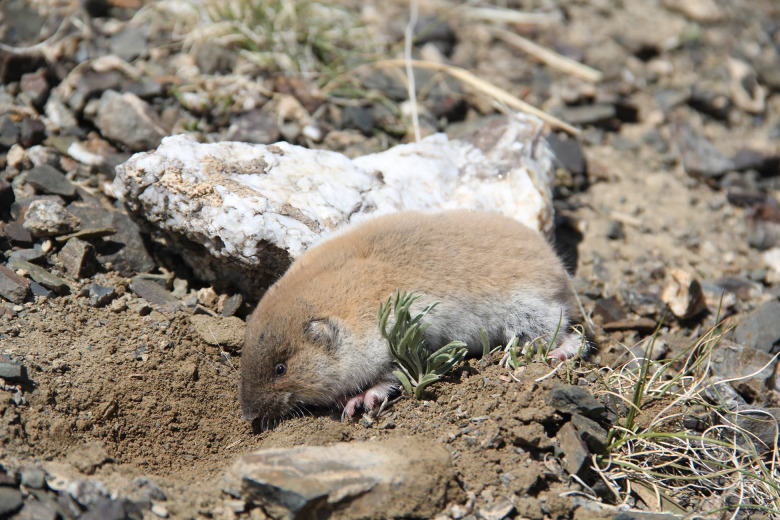
Digital image of the symbiotype host *Ellobius tancrei* Blasius 1844 collected at Baitag Bogd, Hovd province, Mongolia.

During our NSF-funded field expeditions to Mongolia (1999–2012), several cestodes were recovered at necropsy from individuals of *Ellobius tancrei* Blasius, 1884 (Rodentia: Arvicolinae) (Fig. 1) which is the only subterranean species of the sub-family Arvicolinae thus far recorded in Mongolia (Wilson *et al.*, 2017). In addition to the mole-voles, also occurring in isolated populations throughout the area are *Myospalax aspalax* Pallas, 1776 and *Myospalax psilurus* Minle-Edwards, 1874, subterranean rodents of the family Spalacidae. These rodents occupy suitable habitats in north-central and north-eastern Mongolia, respectively (Batsaikhan *et al.*, 2014). Only a single helminth parasite, *Ascarops strongylina* (Rudolphi, 1819) species has been reported from *M. psilurus* collected from eastern Mongolia (Ganzorig *et al.*, 1999).

At the current time, five species of *Ellobius* Fischer, 1814 have been recorded from the Palearctic region with species occupying suitable habitat as far west as the southern Ukraine and as far east as south-central Mongolia and north-central China (Wilson *et al.*, 2017). Current knowledge of the mammal fauna of Mongolia shows that only a single species of *Ellobius* is known (Musser and Carleton, 2005; Tinnin *et al.*, 2011). The distribution of *E. tancrei* within Mongolia extends from the south-eastern Gobi-steppe region through the central steppe grasslands and the Khangai mountains through the north-western regions of the country. Habitat types consist of relatively hard but friable soils in mountain steppe, dry steppe and semi-desert zones (Batsaikhan *et al*., 2014).

Interestingly, before our expeditionary work in Mongolia, cestodes of the genera *Echinococcus*, *Taenia*, *Hymenolepis*, *Mesocestoides*, *Nomadolepis* and *Catenotaenia* had been reported from the species of *Ellobius* only by Tokobaev (1960) from the Kyrgyz Republic, by Zanina and Tokobaev (1962) from Tajikistan and by Alfonso *et al.* (2015) from Sary Mogol, Alay valley, Kyrgyzstan. In 2015, work by a field party from the Museum of Southwestern Biology (MSB) reported *A. microtis* from *Microtus gregalis* (Pallas, 1779), *A. gulyaevi* Makarikov *et al.* 2013 from *Myodes rufocanus* (Sundevall, 1846) and *A. macrocirrosa* Makarikov *et al.* 2011 from *Myodes rutilus* (Pallas, 1779), all were collected from Uvs province in north-western Mongolia (Haas *et al*., 2020), which makes the current report the fourth occurrence record of *Arostrilepis* tapeworm species found from Mongolia.

**Fig. 2.**
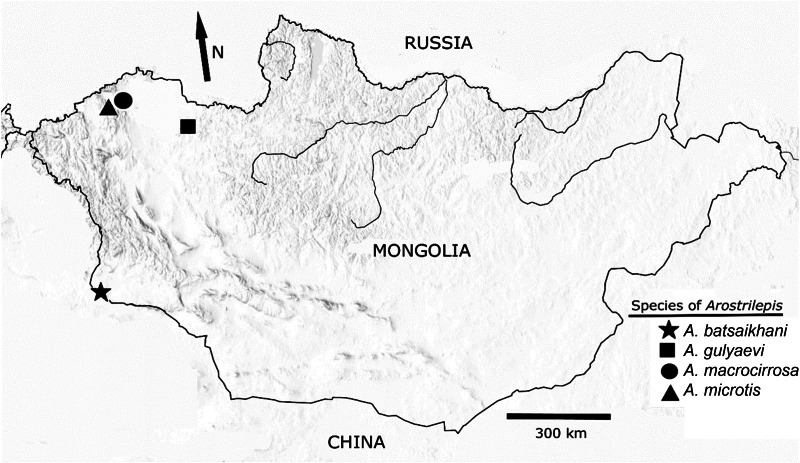
Map of Mongolia showing collection localities of four species of *Arostrilepis* from arvicoline rodents through the western part of the country. Note that collections were made from several dozen separate localities spanning the western half of Mongolia and these four records represent the only places where the cestodes were found.

The most important morphological synapomorphies for species included in the genus *Arostrilepis* (Cestoda: Hymenolepididae) are: cirrus with long spines, lack of any kind of both a rostellum or apical organ, oblong eggs with embryos pointed or tapered on the ends and delicate minute embryonic hooks (see definitions of hymenolepidid eggs in Ubelaker, 1980). Species of *Arostrilepis* have a Holarctic distribution, currently with 15 species reported to occur in 27 species of mammals between latitudes 36°S and 75°N (see Fig. 2). At the present time, arvicoline rodents serve as definitive hosts for more than 80% of the species of these cestodes (Makarikov *et al*., 2020), and the number is increasing.

The first species known from this group was *Arostrilepis horrida* (Linstow, 1901) which was initially described as *Taenia horrida* Linstow, 1901 from *Rattus norvegicus* (Berkenhout, 1769) (Rodentia: Muridae) from the Lower Saxony (Niedersachsen) area of Germany (Linstow, 1901) and subsequently placed in the genus *Hymenolepis* Weinland, 1858. Makarikov *et al.* (2012) described two new species of *Arostrilepis* from disparate rodent groups including *Peromyscus* Gloger, 1841, a member of the widespread new-world family Cricetidae and from a species of *Thomomys* Wied-Neuwied, 1839 of the mostly Nearctic Geomyidae. More recently, from the Appalachian mountains area of eastern North America, Makarikov *et al.* (2020) reported two new species of *Arostrilepis*, including one from jumping mice, *Napaeozapus insignis* (Miller, 1891), of the Holarctic family Dipodidae, a heretofore unreported host group for these cestodes, and the other from a southern red-backed vole, *Myodes gapperi* (Vigors, 1830).

Almost all tapeworms have complex life-histories that require multiple hosts with specific biological, physiological and linked ecological processes that must be perfectly aligned for them to infect these hosts successfully and then, to be transmitted to the next trophic level. Unfortunately, very little is known about the natural life-cycles of the species of *Arostrilepis* even though a proven intermediate host of at least one species includes springtails (Hexapoda: Collembola) (see Smirnova and Kontrimavichus, 1977). Springtails are speciose, globally diverse, omnivorous, free-living animals that live in moist habitats, and relatively recently, an experimental study on the development of metacestode stages of *A. microtis* in springtails has been published (Ishigenova *et al*., 2018). Even with these new data, general information about the natural intermediate hosts, life-cycles and ecology of most species of *Arostrilepis* is sparse at this time, and much more ecological information is needed to discover the intermediate hosts used by these cestodes throughout their ranges. The current paper is a continuation of the process of the documentation part of the DAMA protocol that was described by Brooks *et al.* (2014).

## Materials and methods

In the field in Mongolia, mole-voles were captured using Victor MaCabee™ gopher traps (Victor Pest Management LLC, NY, USA) that were placed in active burrow systems that had evidence of freshly expulsed and mounded soil; these traps euthanize the animal instantly when the vole enters the trap. Our experience shows that the voles appear to work or burrow almost continuously over a 24 h period, so traps were checked at various intervals during both day and night. Elevations from where mole-voles were collected in Mongolia range from about 800 m above sea level (masl) at the lowest in the south-eastern grasslands of the Gobi up to approximately 3000 (masl) in the south and west of the country (Batsaikhan and Tinnin, 2016).

At time of collection, each individual mammal was placed in a single use plastic bag and transported to the field-lab where each specimen was provided a field collection number, standard measurements were taken and tissues were preserved for future study following Yates *et al.* (1996) and Galbreath *et al.* (2019). Specimens were then necropsied, and internal parasites were collected and processed by standard methods following Gardner and Jiménez-Ruiz (2009).

For the study of the morphological characters of these cestodes, we followed the methods outlined by Gardner and Jiménez-Ruiz (2009) with modifications as follows. Tapeworm strobilae discovered at necropsy were relaxed for 20 min in stream water immediately after extraction from the host intestine. They were subsequently killed and fixed in a hot 10% aqueous formalin solution, placed in a Wheaton™ snap cap vial (Fischer Scientific, Pittsburgh, PA, USA), a label was added indicating host, locality, fixative and other data, sealed and transported to the HWML where they were stored in the same solution until study.

In the laboratory, whole mounts were stained with Semichon's acetic carmine, dehydrated in an ethanol series, cleared in cedarwood oil or terpineol, washed briefly in xylene and mounted permanently on microscope slides in Damar gum under a No. 1 cover glass.

For molecular investigation, a part of each individual specimen was removed before fixation and stored for future molecular analysis. Specifically, several gravid, but not terminal, proglottids were removed and either snap-frozen in liquid nitrogen or preserved in 95% ethanol and were subsequently frozen at −80°C in the Parasite Genomic Research Facility in the Manter Laboratory. Note that the terminal gravid proglottids should not be used for molecular study because it is necessary to examine the terminal proglottids on complete specimens to determine the anapolytic or apolytic state. It is also important to know how many proglottids were removed from individual specimens to afford an accurate count of total number of proglottids on a strobila.

**Fig. 3.**
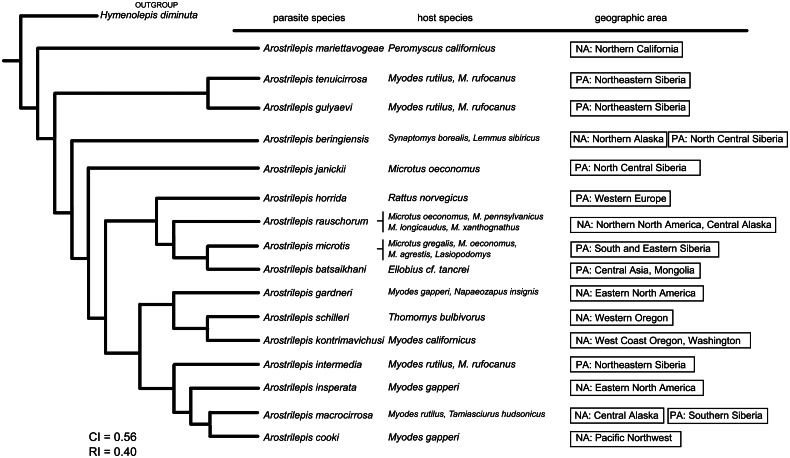
Total evidence phylogenetic tree of all known species of *Arostrilepis*. The tree was constructed using the phylogenetics program TNT 1.5 (Goloboff and Catalano, 2016). Data used to construct the tree included morphological characters (both qualitative and quantitative) and 516 base pairs of DNA from cytochrome-b. *Hymenolepis diminuta* was used as an outgroup.

All parasite specimens studied were deposited in the HWML, and museum catalogue numbers were assigned to each specimen. Specimens were studied with a Zeiss Axiophot™ microscope (Carl Zeiss Microscopy, LLC, White Plains, NY, USA) using both bright-field and Normarsky™ illumination. Images were prepared using Zeiss AxioVision™ (V4.6.3.0) software (Carl Zeiss Microscopy) and Adobe Photoshop CS5™ (Adobe LLC, Damascus, OR, USA). Line drawings (see Fig. 3) were prepared directly from images captured with the Zeiss microscope using the layers function in Photoshop and lines were traced using a stylus equipped Wacom-Intuos™ tablet (Wacom Technology Corporation, Portland, OR, USA). Measurements of organs in mature segments were taken from the last five mature segments, which are the segments immediately anterior to the segment in which eggs begin to appear in the developing uterus. To ensure normality of measurements and to enable the analysis in TNT, all mensural values were log-transformed in either SAS 9.4 or Microsoft Excel. In the description, all measurements are given in micrometres unless otherwise indicated.

For analysis of DNA sequence from an individual cestode, frozen material of specimens stored in the HWML Parasite Genomic Research Facility (PGRF) was used. Non-terminal posterior proglottid tissue samples were used for DNA extraction using Qiagen™ DNeasy Tissue Kits^®^ (Qiagen, California, USA). *Cytochrome-b* (*cytb*) was amplified by PCR using the forward primer HYM01 (5′ATTGTGGTTYTGTTGAATGC) and reverse primers HYM08 (5′GGGATTTTTACTA CCACTCTTGTGC), HYM22 (5′ AAGAAACCGAAAACAATGGAT) and HYMLEM02 (5′CCCACAATAGCAAAYCCCAARCATACATG) (Makarikov *et al.*, 2013). All existing *cytb* sequences stored in GenBank for *Arostrilepis* were downloaded and aligned with Clustal Omega to estimate the extent of evolutionary divergence using genetic distances with the *cytochrome-b* sequences among *Arostrilepis* species for which molecular data are available, we included 516 positions from the dataset, and we analysed the matrix of DNA sequences (which included 11 species: see Table 1) with MEGA X (Kumar *et al*., 2018; Nei and Kumar, 2000). Genetic distance analyses were conducted using the Maximum Composite Likelihood model (Tamura *et al*., 2004) and rate variation among nucleotide sites was modelled with a gamma distribution (shape parameter = 1). For this part of the analysis, all positions containing gaps and missing data were eliminated (complete deletion option).

**Table 1.** Estimates of genetic distance among the species of the genus *Arostrilepis* using 12 cytochrome-b gene sequences with a total of 516 positions using MEGA-X (Kumar *et al*., 2018)

	*Arostrilepis* species	1	2	3	4	5	6	7	8	9	10	11
1	*A. batsaikhani* n. sp. (MW645238)											
2	*A. insperata* (MN019667)	0.134										
3	*A. gardneri* (MN019659)	0.088	0.154									
4	*A. gulyaevi* (JX392029)	0.104	0.141	0.091								
5	*A. cooki* (JX392032)	0.119	0.121	0.135	0.148							
6	*A. rauschorum* (JX392037)	0.109	0.152	0.047	0.077	0.133						
7	*A. macrocirrosa* (JX841310)	0.134	0.190	0.106	0.120	0.179	0.108					
8	*A. tenuicirrosa* (JX126911)	0.095	0.132	0.110	0.105	0.124	0.118	0.150				
9	*A. intermedia* (JX392042)	0.098	0.153	0.048	0.088	0.126	0.064	0.120	0.118			
10	*A. microtis* (JX392045)	0.041	0.122	0.096	0.107	0.123	0.127	0.160	0.096	0.101		
11	*A. beriengiensis* (JX392048)	0.078	0.138	0.093	0.103	0.133	0.098	0.122	0.103	0.098	0.092	
12	*A. janickii* (JX392049)	0.111	0.152	0.135	0.142	0.101	0.142	0.181	0.128	0.136	0.130	0.151

Evolutionary divergence was estimated using the Maximum Composite Likelihood model (Tamura *et al*., 2004). GenBank accession numbers are provided after each species name.

For the phylogenetic analysis of the total evidence data matrix (see Supplementary material), we used the options: new technology search and implicit enumeration in the program TNT, version 1.5 (available for free use *via* sponsorship of the Willi Hennig Society) (see Goloboff and Catalano, 2016). To insure that we used as many data as possible for the phylogenetic estimations, we employed a total evidence approach (Kluge, 1989) to develop and analyse our dataset which consisted of morphological continuous-mensural, discrete-qualitative (Weaver *et al.*, 2016) and DNA sequence characters.

All mammal specimens collected during the MVPP, including individuals of *Ellobius* obtained during this study, have been deposited in the Division of Mammals, MSB, University of New Mexico, Albuquerque, New Mexico. All parasite samples were deposited in the Harold W. Manter Laboratory of Parasitology, University of Nebraska State Museum, Lincoln, Nebraska as voucher and type specimens and all frozen parasite specimens are stored in the freezers of the HWML-PGRF. Genetic data for the parasites studied herein were deposited in GenBank, and tissues of these parasites are stored in the freezers of the parasite genomic research facility in the Manter Laboratory. The current paper is a continuation of the process of the documentation part of the DAMA protocol that was described by Brooks *et al.* (2014).

## Results

The collection locality of the specimens used in this study was the northeast foothills of Baitag Bogd mountain, part of the high Asian Altai Mountain range, located in Hovd province in south-western Mongolia (see Fig. 2). During our work in Mongolia, 103 individuals of *E. tancrei* were collected and we found two infected with an undescribed species of cestode from a single locality.

Following is the description of a new species of *Arostrilepis*. All measurements are given in micrometres unless otherwise noted. *N* is the number of individual morphological characters measured. In the following description, the range of minimum to maximum measurement values is given, and the mean value is separated by a comma followed by standard deviation in parentheses. Measurements of organs in mature segments were taken from the last mature segment and the four segments anteriad, the last mature segment was that one segment immediately anterior to that in which eggs were observed in the developing uterus.

### Description

#### *Arostrilepis batsaikhani* n. sp.

**Fig. 4.**
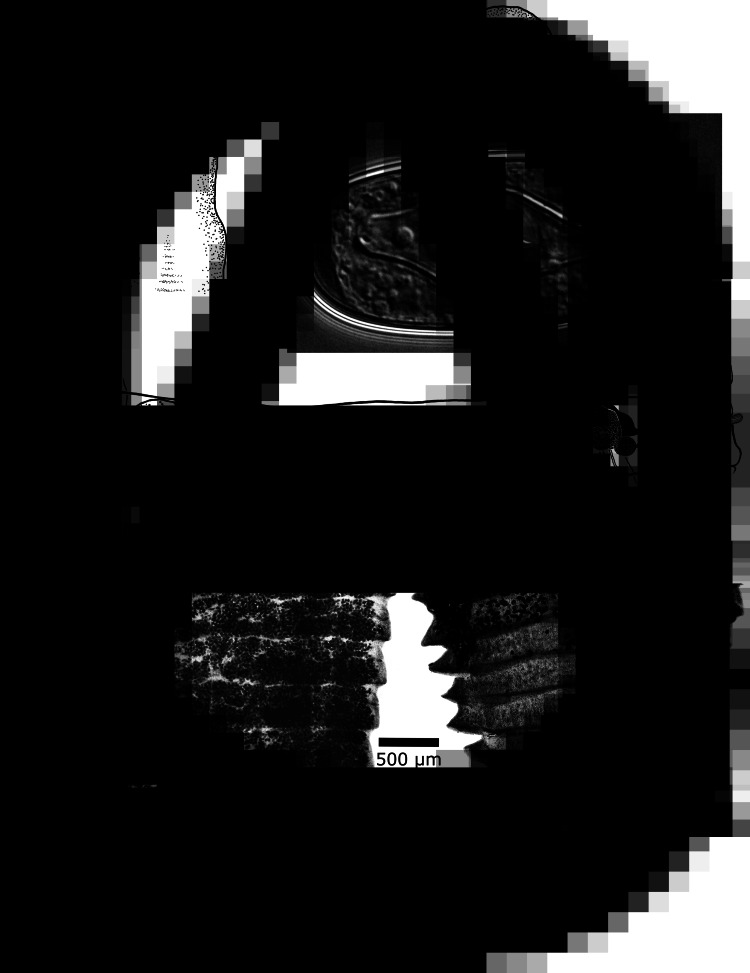
(A–F) Line drawings and images of *Arostrilepis batsaikhani* n. sp. (A) Scolex, showing suckers and lack of apical organ. (B) Drawing of embryo removed from the egg shell showing larval hooks. (C) Digital image of egg showing diagnostic embryophore with bilaterally attenuated shape. (D) Line drawing of the last mature proglottid, dorsal view. (E) Digital image of gravid proglottids showing uterus filled with eggs in sub-terminal proglottids. (F) Digital image of sub-terminal proglottids filled with eggs and terminal proglottid with lack of eggs.

Four specimens were studied for the following description, not all characters visible in each specimen (see Fig. 4A–F); therefore, the number of characters measured is noted as *n*. Scolex unarmed (see Fig. 4A), *n* = 4, 151–272, 208 (44) long by *n* = 4, 170–386, 276 (102) wide. Suckers, *n* = 8, 106–206, 155 (33) long by *n* = 8, 68–176, 125 (44) wide. Neck, *n* = 4, 98–118, 1094 (78) long by *n* = 4, 108–275, 191 (68) wide. Apical organ (rostellum) not present. Strobila craspidote, *n* = 3, 97–143, 123.6 (24) mm long by *n* = 4, 2–3126, 2.53 (0.46) mm in maximum width. Minimum number of proglottids is 1350 (excluding 10 proglottids taken for gene sequencing). Mature proglottid, *n* = 30, 154–255, 198 (31) long by *n* = 30, 1116–1682, 1355 (164) wide (see Fig. 4D). Gravid proglottids *n* = 15, 292–447, 370 (45) long by *n* = 15, 2872–3125, 3003 (91) wide (see Fig. 4E). Proglottids wider than long. Specimens used in this description appear to be anapolytic (see Fig. 4F). In the holotype specimen, it is clear that the eggs have passed out of the last segments of the strobila before the spent segments have detached. However, in the four other individuals, this character was not observed because the posterior segments were cut-off for molecular analysis. Strobilar margins serrate, with intersegmental boundaries well defined in both mature and gravid proglottids.

Genital pores unilateral, dextral. Cirrus *n* = 24, 29–58, 45 (8) long by *n* = 24, 12–21, 16 (3) wide. Antiporal side of cirrus sac does not overlap osmoregulatory canals. The cirrus sac piriform, *n* = 22, 102–170, 134 (19) long by *n* = 22, 36–43, 36 (4) wide. Cirrus spines *n* = 50, 4–5, 4 (0.2). Internal seminal vesicle *n* = 20, 48–109, 69 (14) long by *n* = 20, 28–50, 39 (6) wide. External seminal vesicle *n* = 20, 150–255, 202 (24) long by *n* = 20, 53–98, 71 (10) wide. Testes relatively large equal in size, a single testis may extend from anterior to posterior margins of segment, *n* = 40, 138–249, 180 (29) long by *n* = 40, 123–209, 161 (23.06) wide. Ovary *n* = 30, 116–260, 177 (47) long by *n* = 30, 282–499, 378 (60) wide, arranged linearly with two on antiporal side of ovary and one on poral side of ovary. In rare cases, all three testes may occur on antiporal side of ovary. Vitelline gland *n* = 30, 58–119, 90 (16) long by *n* = 30, 97–212, 146 (31) wide. Seminal receptacle *n* = 30, 256–505, 349 (67) long by *n* = 30, 29–69, 45 (11) wide. Uterus saccular, developing as a transverse tube beginning centrally and slightly anterior in segment and extending laterally as it develops in its saccular shape. Developing uterus crossing both dorsal and ventral excretory canals laterally. Uterus passing dorsally to the dorsal and ventral excretory canals. Also, in developing segments when uterus first appears it extends to the level of the distal end of the cirrus. As eggs begin to appear and mature, the uterus expands, eventually filling the whole segment well-overlapping the lateral excretory canals on both sides of the strobila. Eggs with relatively smooth eggshells (Fig. 4C), *n* = 40, 22–35, 28 (3) long by *n* = 40, 39–54, 47 (3) wide. Developing eggs appear before the uterus reaches lateral margins of segments. Embryophore *n* = 40, 9–13, 10 (1) long by *n* = 40, 21–35, 30 (3) wide. Oncosphere size *n* = 40, 6–9, 7 (1) long by *n* = 40, 8–15, 10 (3) wide. Hooks in onchosphere embryo delicate (Fig. 4B), lengths of hooks *n* = 31, 6–7, 6 (0.2). From each egg, morphological characters of the hooks are as follows: lateral hook pairs dimorphic consisting of one small or more delicate hooks and one more robust hook with a larger guard and slightly thicker blade. Middle pair of hooks both delicate not dimorphic.

#### Taxonomic summary

Symbiotype (see Frey et al., 1992): *Ellobius tancrei* Blasius, 1884, MSB, Division of Mammals, catalogue no. MSB267768, field collection number NK224165, collector: Terry R. Haverkost, collector number TRH1552.

Date of collection: 16 July, 2012.

Holotype: Harold W. Manter Laboratory: HWML216390.

Paratypes: HWML216391, HWML216392, HWML216393, HWML216394.

Site of infection: Small intestine, duodenum.

Type locality: Buduun Hargait Gol, North side of Baitag Bogd, Bulgan soum, Hovd province, Mongolia (45°15′47.1240″S, 90°57′06.0840″W, 1992 m – taken with GPS).

Etymology: This species was named in honour of Batsaikhan Nyamsuren, Professor of Zoology, National University of Mongolia, in recognition of his years of enduring commitment to research in biodiversity and conservation biology in Mongolia.

Prevalence at type locality: 2/10 = 20%.

Infection intensity: Four individuals in one host.

GenBank accession number: MW645238.

Life cycle: The life cycle of this species is unknown.

#### Differential diagnosis

At the current time, 15 species of *Arostrilepis* are known only from rodents in the Northern Hemisphere. *Arostrilepis batsaikhani* n. sp. represents the 16th species in this genus and shares a common ancestor with *A. microtis*, a species with a wide host and geographic range, having been reported from both *Microtus oeconomus* (Pallas, 1776) and *M. agrestis* (Linnaeus, 1761) in the Palearctic. Previous studies have shown that the genetic distance for *Arostrilepis* tapeworms recognized as distinct species based on Cytb locus ranges from 4 to 15% (Makarikov *et al.*, 2013). *Arostrilepis batsaikhani* n. sp. can be recognized as distinct from *A. microtis* by a genetic distance value of 4.1% and the following morphological characters: having larger testes, smaller cirrus sac, cylindrical cirrus and larger cirrus spines (see Table 2).

**Table 2.** Species of *Arostrilepis* and their morphological characters (measurements in micrometres except where otherwise indicated)

Arostrilepis species	Strobila length (mm)	Strobila width (mm)	Scolex width	Suckers	Testes	Cirrus sac	Cirrus	Spines
*A. batsaikhani* n. sp.	97–143	3.12	170–386	106–206 × 68–176	138–249 × 123–209	102–170 × 36–43	29–58 × 12–21	4–5
*A. beringiensis*	100–125	1.4–1.6	230–320	170–270 × 120–160	100–185 × 80–142	95–140 × 25–36	33–44 × 10–12	2.2–2.7
*A. cooki*	150	1.25–1.4	280–372	162–238 × 124–195	115–175 × 85–125	190–218 × 35–48	88–109 × 19–24	3.3–4
*A. gardneri*	49–76	1.0–1.2	275–394	150–171 × 85–135	85–125 × 55–90	193–230 × 33–41	74–94 × 12–14	2.8–3.3
*A. gulyaevi*	180–300	1.9–3.4	250–350	177–253 × 135–185	173–375 × 95–216	192–235 × 32–45	53–72	4–4.5
*A. horrida*	–	1.87–1.93	270–300	133–145 × 128–134	220–290 × 50–70	240–270 × 30–40	88–94 × 6–10	Up to 4.5
*A. insperata*	127–151	1.2–1.9	200	110–125 × 85–88	168–220 × 85–110	225–265 × 48–61	100–110 × 26–31	3.2–3.7
*A. intermedia*	100–150	1.35–1.80	290–320	200–250 × 130–150	145–210 × 70–140	180–210 × 28–40	80–98 × 14–20	3.5–4.0
*A. janickii*	50–80	1.00–2.00	190–240	110–175 × 85–120	95–175 × 50–115	140–177 × 31–42	60–82 × 13–17	3.5–4.3
*A. kontrimavichusi*	65–100	0.75–1.00	173–275	110–140 × 77–118	110–175 × 85–116	148–184 × 31–45	49–65 × 11.5–14	2.8–3.3
*A. macrocirrosa*	116–170	0.9–1.7	290–320	160–200 × 140–170	110–170 × 80–130	195–230 × 35–45	100–128 × 27–34	3.5–4
*A. mariettavogeae*	95–120	1.1–1.3	300–380	140–175 × 110–125	175–246 × 120–165	120–145 × 30–40	30–42 × 5–10	1.5–1.8
*A. microtis*	–	3.0–3.65	220–300	160–190 × 120–150	160–190 × 180–220	220–250 × 45–55	75–85 × 20–22	3–4
*A. rauschorum*	120–185	2.4–3.8	240–300	130–190 × 120–155	130–252 × 110–194	210–242 × 31–42	77–92 × 18–23	3.5–4
*A. schilleri*	67	0.90–0.98	230–250	140–176 × 115–140	110–183 × 70–112	95–123 × 22–31	61–74 × 17–21	3.4–3.7
*A. tenuicirrosa*	–	1.7–2.3	280–360	150–180 × 110–140	200–300 × 140–170	175–225 × 35–45	64–71 × 6–12	2–2.5

Arostrilepis species	Ovary width	Vitellarium	Seminal receptacle	Egg	Oncosphere	Embryophore	Embryonic hooks	Sources
*A. batsaikhani* n. sp.	282–499	58–119 × 97–212	256–505 × 29–69	22–35 × 39–54	6–9 × 8–15	9–13 × 21–35	6–7	Current study
*A. beringiensis*	280–380	60–100 × 130–180	100–180 × 30–40	30–36 × 48–56	8–10 × 13–15	10–14 × 31–36	5–5.7	Makarikov and Kontrimavichus (2011)
*A. cooki*	305–410	60–117 × 115–172	80–122 × 40–77	31–38 × 58–72	13–17 × 18–23	17–20 × 40–48	7.5–8.3	Makarikov *et al.* (2013)
*A. gardneri*	220–290	74–106 × 105–126	140–195 × 40–67	28–39 × 50–65	12–17 × 16–25	16–23 × 42–52	7–8	Makarikov *et al.* (2020)
*A. gulyaevi*	575–715	100–142 × 215–386	150–340 × 30–80	30–37 × 58–68	13–15 × 16–19	16–20 × 40–49	7.2–8	Makarikov *et al.* (2013)
*A. horrida*	620–690	35–44 × 240–270	250–370 × 37–55	30 × 53	10 × 13	11 × 40	–	Makarikov *et al.* (2011)
*A. insperata*	380–450	95–110 × 202–240	169–240 × 58–73	32–40 × 50–56	14–18 × 15–20	16–22 × 34–42	7.5–8	Makarikov *et al.* (2020)
*A. intermedia*	300–460	65–88 × 130–200	110–175 × 35–65	36–42 × 60–65	15–20 × 20–25	18–22 × 42–45	7.5–8.2	Makarikov and Kontrimavichus (2011)
*A. janickii*	230–415	45–80 × 95–185	87–162 × 47–70	27–36 × 43–52	10–11 × 14–15	11–15 × 34–40	6.5–7.7	Makarikov and Kontrimavichus (2011)
*A. kontrimavichusi*	185–310	74–120 × 116–153	50–115 × 35–55	26–31 × 39–45	10–11 × 12–15	11–14 × 25–31	6.5–7.5	Makarikov and Hoberg (2016)
*A. macrocirrosa*	250–430	73–86 × 120–190	120–190 × 55–78	33–45 × 52–63	17–20 × 25–32	20–24 × 40–45	8–8.5	Makarikov *et al.* (2011)
*A. mariettavogeae*	215–390	40–72 × 120–166	105–195 × 18–33	23–27 × 33–38	9–12 × 12–15	10–14 × 23–27	7.2–8.2	Makarikov *et al.* (2012)
*A. microtis*	450–620	80–100 × 160–230	550–840 × 60–85	22–25 × 45–48	7.5–8.5 × 14–15	–	–	Gulyaev and Chechulin (1997)
*A. rauschorum*	455–585	75–123 × 145–240	165–280 × 50–92	22–35 × 50–68	11–17 × 15–22	14–20 × 38–47	7.5–8.3	Makarikov *et al.* (2013)
*A. schilleri*	210–310	45–70 × 80–125	75–105 × 25–42	24–31 × 40–48	9–13 × 10–15	12–16 × 23–31	7.7–9	Makarikov *et al.* (2012)
*A. tenuicirrosa*	400–570	80–110 × 140–200	175–290 × 35–50	30–34 × 50–57	14–17 × 18–22	18–22 × 35–44	7–8	Makarikov *et al.* (2011)

Table shows character length by width for the summary of each measurement.

Further, *A. batsaikhani* can be recognized as distinct from other species in the genus by the following morphological characters: the arrangement of testes for *A. batsaikhani* is strictly linear, which distinguishes *A. batsaikhani* from all described species of *Arostrilepis* known up to the present time except *A. microtis* and *A. rauschorum*. Further, *A. batsaikhani* can be differentiated from *A. rauschorum* by having a smaller cirrus sac, a smaller cirrus and larger cirrus spines.

#### Additional considerations of *Arostrilepis* evolution

To understand the degree of genetic differentiation of *A. batsaikhani* relative to the other species in the genus, a genetic distance analysis was conducted using *cytochrome-b* gene sequence data. Table 1 shows the genetic affinities of *A. batsaikhani* to the rest of the species in the genus for which data on *cyt-b* were available. Finally, combining both morphology and available molecular cytochrome-b data for all known species enabled us to create a phylogeny to examine the placement of *A. batsaikhani* relative to the rest of the described species of this genus, see Fig. 3. Data relative to this tree included, for 17 species, 516 base pairs of *cytochrome-b* DNA, 35 quantitative characters and 18 qualitative characters that were concatenated and run as a single input file to TNT. Our analysis produced a single most parsimonious tree with a consistency index of 56% and retention index of 40%. Our tree (Fig. 3) shows that *A. batsaikhani* n. sp. is well-embedded within the in-group, with *A. microtis* as its sister species.

## Discussion

The current study describes the 16th species in the genus *Arostrilepis* which includes a new host record and raises the number of species of known rodent hosts to 28 (see Table 3). The first record of any species of *Arostrilepis* reported from a subterranean rodent was *A. schilleri* from *Thomomys bulbivorus* (Richardson, 1829) from south-east of Corvallis, Oregon by Gardner (1985), who did not recognize that the species was new at the time (see the correction of this error in Makarikov *et al.*, 2012).

**Table 3.** Species of *Arostrilepis* and their hosts with zoogeographic regions of occurrence

*Arostrilepis* species	Host species	Geographic records
*A. gulyaevi* Makarikov *et al.* 2013	*Myodes rufocanus* (Sundevail, 1846), *My. rutilus* (Pallas, 1779)	Holarctic
*A. cooki* Makarikov *et al.* 2013	*My. gapperi* (Virogs, 1830)	Nearctic
*A. microtis* Gulyaev and Chechulin, 1997	*Mi. oeconomus* (Pallas, 1776), *Mi. gregalis* (Pallas, 1779), *Microtus agrestis* (Linnaeus, 1761)	Holarctic
	*Mi. arvalis* (Pallas, 1778), *Mi. maximowiczii* (Schrenck, 1859)	
*A. macrocirrosa* Makarikov *et al.* 2011	*My. rutilus*, *Tamiasciurus hudsonicus* (Erxleben, 1777)	Holarctic
*A. rauschorum* Makarikov *et al.* 2013	*Mi. oeconomus*, *Mi. pennsylvanicus* (Ord, 1815) *Mi. longicaudus* (Merriam, 1888)	Holarctic
	*Mi. xanthognathus* (Leach, 1815), *Mi. miurus* Osgood, 1901	Nearctic
*A. beringiensis* Gulyaev and Chechulin, 1997	*Lemmus sibiricus* (Kerr, 1792), *My. schisticolor* (Lilljeborg, 1844)	Palearctic
	*Synaptomys borealis* (Richardson, 1828)	Nearctic
	*L. trimucronatus* (Richardson, 1825)	Holarctic
*A. tenuicirrosa* Makarikov *et al.* 2011	*My. rutilus*, *My. rufocanus*	Holarctic
	*My. rex* (Imaizumi, 1971)	Palearctic
*A. intermedia* Makarikov and Kontrimavichus, 2011	*My. rufocanus*	Palearctic
	*My. rutilus*	Holarctic
*A. janickii* Makarikov and Kontrimavichus, 2011	*Arvicola scherman* Shaw, 1801, *A. amphibius* (Linnaeus, 1758)	Palearctic
	*Mi. arvalis*, *Chionomys nivalis* (Martins, 1842), *Mi. multiplex* (Fatio, 1905)	Palearctic
*A. kontrimavichusi* Makarikov and Hoberg, 2016	*My. californicus* (Marriam, 1890)	Nearctic
*A. gardneri* Makarikov *et al.* 2020	*My. gapperi* (Vigors, 1830), *Napaeozapus insignis* (Miller, 1891)	Nearctic
*A. insperata* Makarikov *et al.* 2020	*My. Gapperi*	Nearctic
*A. batsaikhani* n. sp.	*Ellobius tancrei* Blasius, 1884	Palearctic
*A. horrida* Mas-Coma and Tenora, 1997	*Rattus norvegicus* (Berkenhout, 1769)	Palearctic
*A. mariettavogeae* Makarikov *et al.* 2012	*Peromyscus californicus* (Gambel, 1848)	Nearctic
*A. schilleri* Makarikov *et al.* 2012	*Thomomys bulbivorus* (Richardson, 1829)	Nearctic

Up to this point in time, no other species of *Arostrilepis* have been reported from subterranean hosts in the Palearctic; however, we expect that additional sampling of subterranean rodents throughout their ranges will reveal greater diversity of parasites in the future. Finally, there are many *Arostrilepis* samples in museums that remain unidentified and work on these cestodes would serve well to expand the evolutionary scope and future investigations into this interesting group of parasites that would require collecting and analysing the data already existing in museums (Brooks *et al*., 2014).

## Supplementary material

For supplementary material accompanying this paper visit https://doi.org/10.1017/S0031182022000294. click here to view supplementary material
